# The unique role of smartphone addiction and related factors among university students: a model based on cross-sectional and cross-lagged network analyses

**DOI:** 10.1186/s12888-023-05384-6

**Published:** 2023-11-27

**Authors:** Cunjia Liu

**Affiliations:** College of Information and Intelligence, Hunan Agricultural University, Changsha, China

**Keywords:** Smartphone addiction, Cross-sectional network analysis, Cross-lagged panel network analysis, Interaction of Person-Affect-Cognition-Execution model, The network theory of mental disorder

## Abstract

Smartphone addiction is a global problem affecting university students. Previous studies have explored smartphone addiction and related factors using latent variables. In contrast, this study examines the role of smartphone addiction and related factors among university students using a cross-sectional and cross-lagged panel network analysis model at the level of manifest variables. A questionnaire method was used to investigate smartphone addiction and related factors twice with nearly six-month intervals among 1564 first-year university students (*M* = 19.14, *SD* = 0.66). The study found that procrastination behavior, academic burnout, self-control, fear of missing out, social anxiety, and self-esteem directly influenced smartphone addiction. Additionally, smartphone addiction predicted the level of self-control, academic burnout, social anxiety, and perceived social support among university students. Self-control exhibited the strongest predictive relationship with smartphone addiction. Overall, self-control, self-esteem, perceived social support, and academic burnout were identified as key factors influencing smartphone addiction among university students. Developing prevention and intervention programs that target these core influencing factors would be more cost-effective.

**Supplementary Information:**

The online version contains supplementary material available at 10.1186/s12888-023-05384-6.

## Introduction

### The significance of research problem

With the rapid development of information technology, the Internet plus era has gradually integrated into all aspects of our lives. More and more people are using internet based applications and devices. While providing convenience, the internet can also lead to excessive dependence and even addiction. The research on problematic use of internet includes two aspects, general and specific internet addiction. Specific internet addiction emphasizes focusing on specific activities on the internet, while general internet addiction emphasizes engaging in all daily activities on a particular device or internet [1]. A smartphone is a general term for a type of mobile phone that has an independent operating system, independent running space, and can be installed by users on their own by third-party service providers, and can achieve wireless network access through mobile communication networks [2, 3]. Therefore, smartphone addiction can be seen as a type of general internet addiction [4–7]. The 51st Statistical Report on Internet Development in China released by the China Internet Network Information Centre (CNNIC) shows that as of December 2022, the number of mobile phone Internet users in China reached 6.5 billion, and the proportion of Internet users using smartphone to access the Internet was 99.8 percent [8]. Some scholars have found that smartphone addiction is detected at a high rate among university students, with the rate of smartphone addiction among Chinese university students reaching 21.3% [9], a cross-culturally consistent finding, with 36.4% of university students in South Korea suffering from long-term smartphone addiction [10], and in Saudi Arabia, the rate of smartphone addiction detection among university students is as high as 48.0% [11].

Smartphone addiction, also known as problematic smartphone use, refers to addictive behavior in which individuals are unable to control their smartphone usage, leading to impaired physical, psychological, and social functioning [12, 13]. Chóliz [14] predicted that smartphone addiction would be one of the most significant addictive behaviors in the twenty-first century, making it a hot topic for scholars. Consequently, researchers have conducted numerous studies on the predictors and negative effects of smartphone addiction to provide guidance for prevention and intervention [15]. Ran et al. [16] found that social anxiety is a significant predictor of smartphone addiction in both adolescents and adults. Therefore, it should be considered when designing intervention programs for these age groups.

For studies related to the consequences of smartphone addiction, results show that smartphone addiction leads to reduced sleep quality [17, 18], impaired cognitive functioning [19, 20] decreased academic motivation and consequently lower grades [21, 22], and development of negative emotions such as anxiety, depression, and loneliness [23–26]. In recent years, some researchers have argued that some factors such as self-control and loneliness, which are both antecedents of smartphone addiction, are also affected and are consequences of smartphone addiction [15]. Therefore, research on smartphone addiction should not only focus on exploring the relationship between smartphone addiction and related factors but also investigate their interactions. Network analysis, as an emerging research method, allows for different variables to be placed into the same visual network model to reveal the interactions between variables [27–29]. This paper adopts network analysis approach to explore the interactions between university students' smartphone addiction and related factors. Its aim is to identify the core factors influencing university students' smartphone addiction.

### Theory construction and selection of influencing factors

#### Interaction of Person-Affect-Cognition-Execution model (I-PACE model)

The I-PACE model, proposed by Brand et al. [30], is a process model applicable to various addictive behaviors [31]. It has been widely used in smartphone addiction research in recent years [32–34]. This model states that specific Internet use disorders are the result of the interaction of core individual characteristics (including psychopathology, personality, social cognition, etc.), affective-cognitive responses, gratification, executive and control functions [30]. That is to say, individual characteristics, affective-cognitive responses, gratification, executive and control functions are considered as triggering variables, while specific network usage barriers are considered as outcome variables. It is worth noting that in the I-PACE model, especially in the early stages of addiction formation, the decision behavior of using specific applications or websites may bring short-term positive experiences and satisfaction. Therefore, an individual's current or past mental health status (such as psychological distress) can affect the problematic use of online activities, leading to an increase in screen time and addiction [35–37].

#### The network theory of mental disorders

The Network Theory of Mental Disorders, proposed by Borsboom [27], suggests that mental disorders arise from causal interactions between symptoms through physical, psychological, and social mechanisms. If these interactions are strong enough, psychopathological symptoms will generate a certain degree of feedback and self-sustaining, leading to a disorderly state of the network, namely mental disorder.

For mental disorder interventions, it is important to focus on the status of the core symptoms in the network in order to change the structure of the network distribution. Jones, Heeren, & McNally [38] expanded on this theory by adding that the nodes in the network of mental disorders are not limited to just the symptoms, but that biological, cognitive, or other individual-level processes can trigger mental disorders.

#### Interaction of I-PACE model with the network theory of mental disorder and selection of influential factors

The I-PACE model reveals a wide range of factors affecting smartphone addiction from a process perspective, but some scholars have argued that the model lacks an exploration on the patterns of interactions between the factors [39]. The network theory of mental disorders, which focuses on causal interactions, underpins the idea of this study to incorporate the variables involved in the I-PACE model into the network of mental disorders and to explore the interactions between them. Based on the Interaction of the I-PACE model and combining previous studies, the present author identified the variables social anxiety, self-esteem, and comprehending social support as core individual characteristics [30], fear of missing out, academic burnout, and procrastination behaviours as emotional-cognitive reactive factors affecting smartphone addiction [39–41], gratification as well as executive and control functions chosen for life satisfaction and self-control [30, 42].

### Network analysis

Network analysis is based on the network theory of mental disorder by placing different personality traits, influences, and symptoms, among others, in the same visual network in order to assess the complex relationships and interactions between them [28, 29, 43]. Nodes in a network represent observed variables, and edges connecting nodes represent statistical relationships between observed variables [28]. In recent years, researchers have used network analysis to build networks of relationships between numerous variables or symptoms to explore complex psychological constructions, and this approach has also been widely used in the field of smartphone addiction research. For example, Li et al. [44] used network analysis to understand the interrelationships between misplaced anxiety, smartphone addiction, and social networking site usage. It has also been found that loss of control and overuse are core symptoms of smartphone addiction [45]. Most of these studies are based on cross-sectional data at a given time and can illustrate interrelationships between variables or symptoms, but caution is needed regarding causal inferences. Rhemtulla et al. [46] developed cross-lagged panel network analysis using cross-lagged panel data in conjunction with network analysis. This method can reveal the longitudinal processes that occur within and across structures over time [47]. Meanwhile, cross-lagged panel network can confirm the stronger impact of core nodes on other nodes, indicating the activation role of core nodes in the entire network [48, 49].

### The objective of this study

This study is guided by the I-PACE model and the network theory of mental disorder, and uses cross-sectional and cross-lagged panel network models to analyze university students' smartphone addiction and its related influencing factors. The study examined smartphone addiction and its influencing factors, including personal core characteristics, affective and cognitive responses, gratification, executive and control functions. The main objective of this study is to explore the transverse correlation and vertical prediction relationship between smartphone addiction and related influencing factors among university students. This paper aims to identify the core factors among these related influencing factors through some central indicators, and provide more specific implications for the future intervention of smartphone addiction in university students.

## Methods

### Participants

This study administered a questionnaire survey to first-year students in a specified province of China in early October 2022 and at the end of March 2023. The survey was conducted utilizing cluster sampling based on colleges as the unit of analysis. The present author contacted counsellors from multiple colleges at a university, outlining the study's objectives and distributing electronic advertisements to recruit participants. At the same time, participants will be informed about the reward they will receive for their participation in the study to incentivize them to take part. In subsequent studies, the author contacted participants who took part in the original study using the same manner. All participants completed the survey on the Wenjuanxing platform, a prevalent Chinese survey website. The study requires participants to possess smartphones or computers with internet access and understand the relevant questionnaire's content.

There were 1802 participants participating in the first test and 1678 in the second test, and the present author matched the data obtained from the two administrations based on demographic variables such as the participant's name, date of birth, etc., and excluded the questionnaires with consecutive unchanging responses and too many missing answers. A total of 1564 university students were included in the analysis, including 854 male and 710 female, with a mean age of 19.14 years (*SD* = 0.66). All participants gave their informed consent for inclusion before they participated in the study. The study was conducted in accordance with the Declaration of Helsinki, and the protocol was approved by the Ethics Committee of the university (Number: LL2023060).

### Measurements

#### Smartphone addiction scale for university students

Su et al. [50] developed a smartphone addiction scale suitable for Chinese university students, which has been widely used by Chinese researchers [51, 52]. The scale contains 22 items and 6 dimensions: withdrawal behaviour, salience behaviour, social comfort, negative effects, use of application, and renewal of application. In this study, Cronbach's α was 0.917, 0.943 for both measures.

#### Aitken procrastination inventory

The Chinese version of the Procrastination Behaviour Questionnaire was developed by Aitken et al. [53] and translated and revised by Chen et al. [54], which was used in this study. The scale consists of 19 items, of which 2, 4, 7, 11, 12, 14, 16, 17 and 18 are reverse scored and items are rated using a scale from one (completely non-compliant) to five (fully compliant), with higher total scores indicating higher procrastination behaviour. Cronbach's α was 0.846 and 0.806, respectively.

#### Learning burnout scale

This study used the Learning Burnout Scale for University Students [55] to measure academic burnout among university students, which consists of a 20-item self-report scale with items 1, 3, 6, 8, 11, 13, 15, and 18 are inversely scored. The scale includes three dimensions: dejected, improper behaviour and reduced personal accomplishment. Higher scores indicate higher academic burnout. Each item is assessed on a five-point Likert scale from 1 (completely non-compliant) to 5 (completely compliant). Cronbach's α of the scale in this study was 0.886, 0.835.

#### Self-control scale

Self-control was measured through the Self-Control Scale developed by Tangney et al. [56] and localized and revised by Tan et al. [57]. The scale includes 19 items and 5 dimensions: impulse control, healthy habits, resisting temptation, focused work, and abstinence from entertainment. These items were evaluated using a 5-point Likert scale. Higher scores reflect greater self-control. Cronbach's α was 0.822 and 0.812 for the two time point measures.

#### Fear of missing out scale

The Fear of Missing Out Scale [58] includes 8 items and 2 dimensions: fear of missing out on information and fear of missing out on a situation. Items were scored on a 5-point Likert scale, with higher scores indicating a greater fear of missing out, and Cronbach's α was 0.814 and 0.850 for the two time point measures.

#### Social avoidance and distress scale

In this study, the Social Avoidance and Distress Scale was used to measure the social anxiety of recent university students. Peng et al. [59] revised the scale, which is suited for Chinese university students, contains 15 questions, and uses a 5-point Likert scale. Reverse scored items included: 3rd, 6th, 10th, and 15th. Cronbach's α was 0.853 and 0.839 for the two time point measures.

#### Perceived social support scale

Jiang [60] revised the Collaborative Social Support Scale developed by Zimet [61] and others, which has good reliability and validity among Chinese university students, with 12 items, including 3 dimensions of family support, friend support, and other support, and is a 7-point Likert scale, with Cronbach's α was 0.941 and 0.961 for the two time point measures.

#### The satisfaction with life scale

The Satisfaction with Life Scale was developed by Diener [62], translated and revised by Qiu [63], which was used to measure the life satisfaction of university students. The scale has five items, each of which is scored using a 7-point Likert scale. The Cronbach's α for this scale in this study were 0.874 and 0.876.

#### Self-esteem scale

In this study, the present author used the Self-Esteem Scale developed by Rosenberg [64] and adopted by Zhou [65] to measure the self-esteem level of Chinese university students, which has 10 items with reverse scoring including questions 3, 5, 8, 9 and 10. Cronbach's α was 0.861 and 0.826 for the two time point measures.

### Analytical procedure

Data collected on smartphone addiction and related factors among university students were descriptively analyzed using SPSS 26.0 and correlation heatmaps for each variable were produced using corrplot function package of the R software package (version 4.3.0). Both the cross-sectional network analysis as well as the cross-lagged panel network analysis used the qgraph package for network modelling [66, 67]. All networks are based on the extended bayesian information criterion [54], which employs the graphical least absolute shrinkage and selection operator (GLASSO) to remove weakly connected edges, obtaining low complexity and high accuracy network model [68].

In the cross-sectional network analysis, the present author constructed two networks at T1 and T2, and for a clearer and more intuitive comparison, the present author chose the "circle" distribution to fix the same nodes of the two networks at the same position. For the core nodes, this paper uses strength as a central metric for evaluation. Strength centrality is the sum of the absolute values of the strength of a node's connections to other nodes, indicating the node's direct effect on the other nodes in the network [43], and a high strength centrality indicates that the node is at the core of the network. In the cross-lagged panel network analysis, regularized regression estimation using the R package glmnet [69] was used to construct a cross-lagged panel network with each of the variables at T1 as a predictor variable and the variable at time T2 as an outcome variable. The centrality measures of the variables include In-strength and Out-strength. The In-strength is the sum of the direct influence of a node in T2 by other nodes in T1, and is the extent to which this node is predicted by other nodes in the network. Out-strength, on the other hand, is the sum of the direct influence of a node in T1 on the other nodes in T2, indicating the degree to which that node predicts the other nodes in the network [70, 71].

Finally, the accuracy and stability of the T1 and T2 networks were evaluated using the R function package bootnet [28, 72], respectively, the correlation stability coefficient and 95% confidence intervals (CI) for edge weights derived by using the nonparametric bootstrap method (bootstrap sample of 1000) respectively.

## Result

### Descriptive statistical analysis

**Fig. 1:**
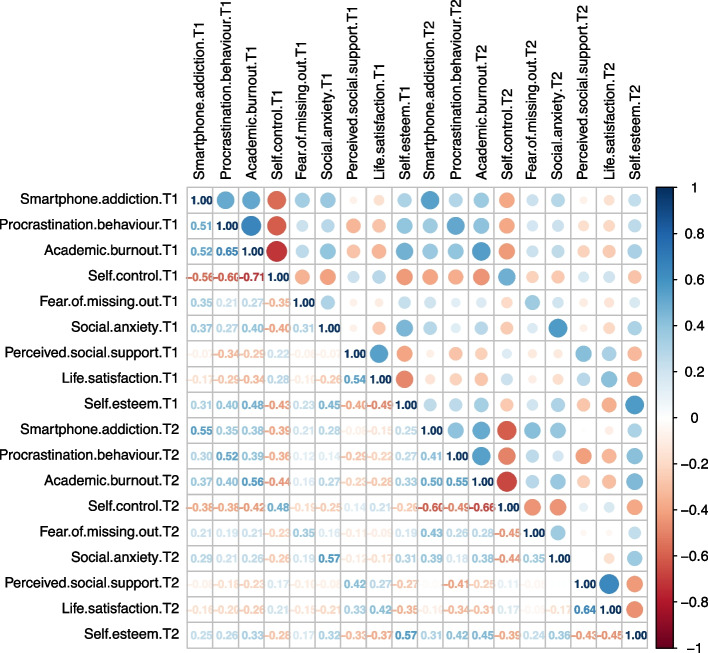
Heatmap of the correlation matrix between smartphone addiction and its influencing factors at T1 and T2 among university students

Table 1 and Fig. 1 display the means, standard deviations, and correlations of all variables in this study. Overall, smartphone addiction among university students was moderate on both measures. The severity of smartphone addiction was positively correlated with procrastination behaviours, academic burnout, fear of missing out, social anxiety, and self-esteem. Self-control, perceived social support, and life satisfaction negatively correlated smartphone addiction.

**Table 1:** Means and standard deviations among university students' smartphone addiction and its influencing factors at T1 and T2

Variable	*M*	*SD*	Variable	*M*	*SD*
Smartphone addiction T1	52.16	13.84	Smartphone addiction T2	52.39	15.49
Procrastination behaviour T1	44.38	9.53	Procrastination behaviour T2	47.52	9.38
Academic burnout T1	53.04	10.83	Academic burnout T2	56.13	9.63
Self-control T1	62.19	9.18	Self-control T2	61.85	8.92
Fear of missing out T1	17.74	5.43	Fear of missing out T2	17.61	5.72
Social anxiety T1	43.75	9.58	Social anxiety T2	43.46	8.87
Perceived social support T1	60.09	13.99	Perceived social support T2	55.70	15.59
Life satisfaction T1	21.49	6.18	Life satisfaction T2	21.50	6.06
Self-esteem T1	26.90	4.97	Self-esteem T2	27.05	4.71

### Cross-sectional network analysis

**Fig. 2:**
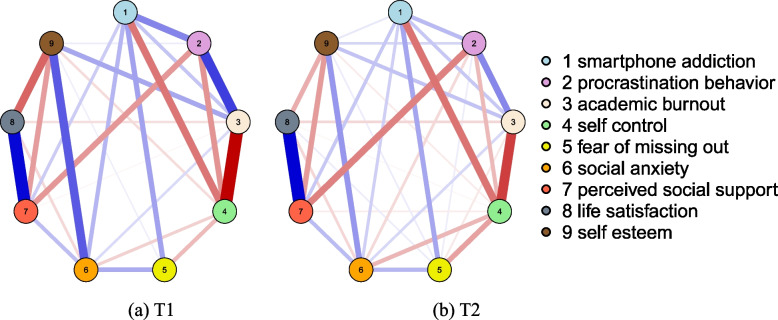
EBICglasso network structure of smartphone addiction and its related factors at T1 and T2 among university students. Note. Blue lines indicate a positive correlation between the variables, while red lines indicate a negative correlation between the variables; "circle" distribution is used

**Fig. 3:**
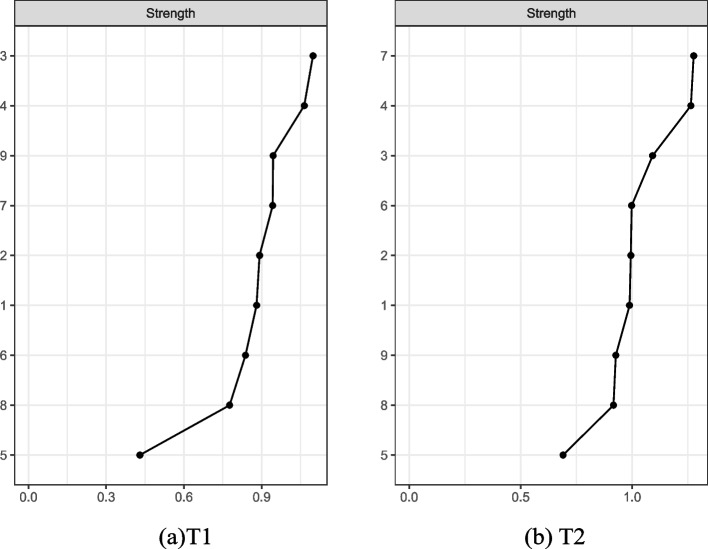
The centrality of intensity on smartphone addiction and its related factors at T1 and T2 among university students. Note. 1 = smartphone addiction; 2 = procrastination behaviour; 3 = academic burnout; 4 = self-control; 5 = fear of missing out; 6 = social anxiety; 7 = perceived social support; 8 = life satisfaction; 9 = self-esteem

The results of the cross-sectional network analysis of smartphone addiction and its associated factors at T1 and T2 are shown in Fig. 2(a) and (b), with strength centrality as in Fig. 3(a) and (b). The results of network stability show that the central stability coefficient of node strength concerning university students' smartphone addiction and its related factors at T1 and T2 is 0.75, and the network of university students' smartphone addiction and its related factors is stable and has a close interaction. In the Fig. 2(a), there are 9 nodes and 26 non-zero edges in the network, node 3 (academic burnout) and node 4 (self-control) are at the center of the network and have relatively high strength centrality (Strength = 1.15, 0.98). The strongest edge strengths (*r* = -0.42) existed between node 3 (academic burnout) and node 4 (self-control). The nodes directly associated with node 1 (smartphone addiction) include node 2 (procrastination behaviour), node 3 (academic burnout), node 4 (self-control), node 5 (fear of missing out), node 6 (social anxiety), and node 7 (perceived social support). Among them, node 4 (self-control) is the most directly associated (*r* = -0.23).

In the Fig.2(b), there are 9 nodes and 32 non-zero edges in the network, node 7 (perceived social support) and node 4 (self-control) have high centrality of strength (Strength = 1.44, 1.37), which is at the center of the network.

### Cross-lagged panel network analysis

**Fig. 4:**
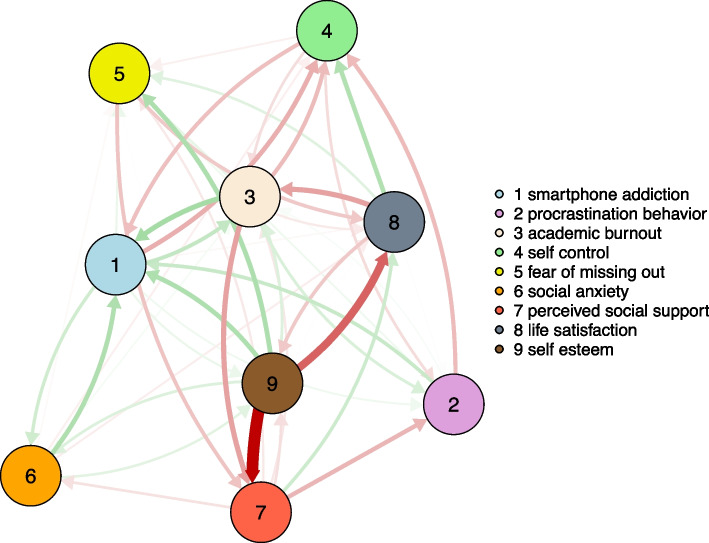
The cross-lagged panel network model on university students' smartphone addiction and influencing factors from T1 to T2, with autoregressive path hidden. Note. Green arrows in the network indicate positive predictions and red arrows indicate negative predictions

Appendix Fig. A1 shows the cross-lagged panel network estimations of smartphone addiction and related factors among university students from T1 to T2. Each variable has a strong autoregression, and node 1 (smartphone addiction) has the largest autoregressive coefficient (*r* = 0.49). To make the cross-lagged paths of the variables clearer, the estimated cross-lagged panel network after hiding the autoregressions is shown in Fig. 4. The results showed a clear correlation between node 1 (smartphone addiction) and node 3 (academic burnout), node 4 (self-control), and node 6 (social anxiety). The nodes that have a direct effect on node 1 (smartphone addiction) are node 2 (procrastination behaviour), node 3 (academic burnout), node 4 (self-control), node 5 (fear of missing out), node 6 (social anxiety), and node 9 (self-esteem), among them the strongest path of relationship existing with node 3 (academic burnout).

**Fig. 5:**
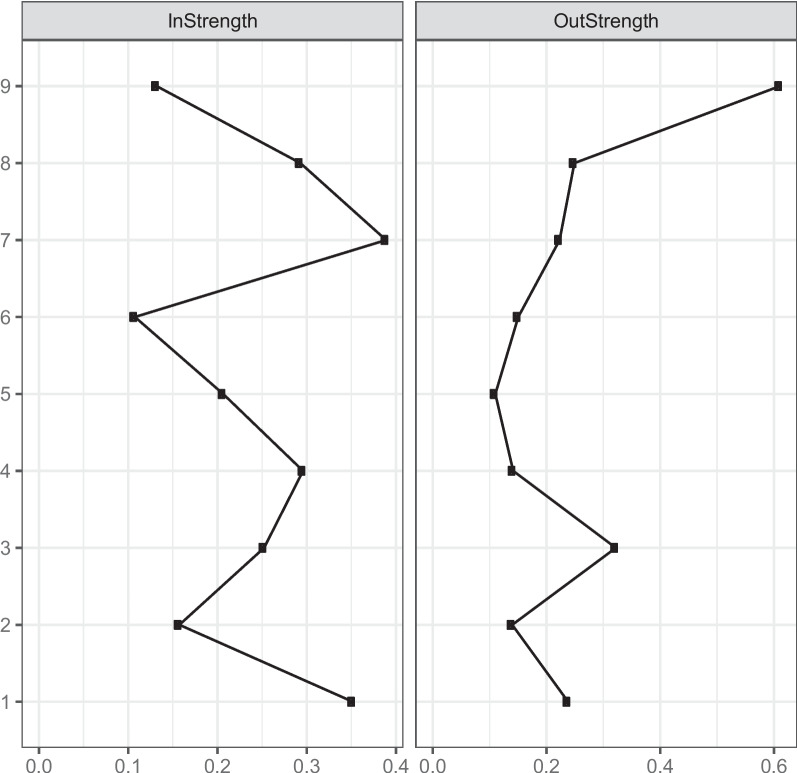
The in-strength and out-strength of the cross-structure of variables in the cross-lagged panel network among university students with smartphone addiction and its correlates from T1 to T2. Note. 1 = smartphone addiction; 2 = procrastination behaviour; 3 = academic burnout; 4 = self-control; 5 = fear of missing out; 6 = social anxiety; 7 = perceived social support; 8 = life satisfaction; 9 = self-esteem

Figure 5 displays the centrality estimations from the cross-lagged panel network model regarding smartphone addiction and its correlated factors among university students from T1 to T2. The results show that node 1 (smartphone addiction), node 7 (perceived social support) and node 9 (self-esteem) are the core nodes in the network. Node 1 (smartphone addiction) and node 7 (perceived social support) have high in-strength inputs and are important outcome variables in the network, and node 9 (self-esteem) has the highest out-strength and is an important predictor variable.

## Discussion

### The correlation between smartphone addiction and influencing factors among university students

The present study, based on network analysis of cross-sectional and longitudinal data, reveals the distinctive role of smartphone addiction and its correlates among university students. Cross-sectional network analysis of T1 and T2 revealed the strongest interrelationship between smartphone addiction and self-control. Previous studies have found that self-control plays a direct or indirect role in the mechanisms of smartphone addiction onset [73–76]. Our study extends these findings and suggests a negative correlation between self-control and smartphone addiction. Self-control is associated with the onset of smartphone addiction, and smartphone addiction can also lead to a decrease in an individual's self-control. The dual-systems theory of self-control [77] and the energy model of self-control [78] support the results of this study. On the one hand, the inhibitory system plays an important role when university students are confronted with the temptation of a smartphone, meaning that high self-control will help individuals resist excessive smartphone use [76]. On the other hand, university students suffering from smartphone addiction for a long period of time can lead to depletion of psychological resources, which ultimately reduces university students' self-control [79].

In addition, academic burnout and self-control were the core factors in the network of smartphone addiction and its correlates among university students in T1. However, in T2, perceived social support and self-control had the greatest influence on the overall network. This suggests that focusing on these core factors changes the structure of the network to a greater extent [43]. It also indicates that cross-sectional networks change over time, leading to different inferences from the core nodes in the network. Further clarification of these findings requires longitudinal data [80].

### The causal relationship between smartphone addiction and influencing factors among university students

In the cross-lagged panel network, the present study focused on the extent to which smartphone addiction was induced by other variables in the network. It was found that procrastination behavior, academic burnout, self-control, fear of missing out, social anxiety, and self-esteem all directly predicted smartphone addiction, consistent with previous studies [26, 81–84]. The present author interpreted this result using the comprehensive path model, which suggests that there are three paths leading to smartphone addiction. The first pathway is the excessive comfort pathway, and it is generally accepted that smartphone addiction occurs more often in individuals with low self-esteem and social anxiety, who lack the appropriate sense of security and use their smartphones excessively as a way of maintaining relationships with others and seeking help. The second pathway is the impulsive pathway, a pathway that argues that smartphone addiction is due to an individual's low impulse control and lack of planning. The third pathway is the open pathway, which explains smartphone addiction as an individual's frequent use of smartphone due to strong social motivation, suggesting that fear of missing out induces smartphone addiction in university students [13, 85, 86]. Additionally, smartphone addiction predicts self-control, academic burnout, social anxiety, and perceived social support in university students, consistent with previous studies. Achangwa et al. [87] found that smartphone addiction among university students negatively affects their physical, mental, and emotional health, academic performance, and social interactions.

Regarding the central role of self-esteem on smartphone addiction among university students, Billieux et al. [12] found that individuals with low self-esteem are more prone to interpersonal distress in real life and have a greater need for emotional security. This need is fulfilled through smartphone communication, leading to smartphone addiction [88]. Therefore, treatment and intervention for smartphone addiction among university students should focus on self-esteem.

### Research significance

This study has theoretical and practical significance for the intervention and treatment of smartphone addiction among university students. Theoretically, it extends the understanding of the interrelationships between smartphone addiction and its correlates using network analysis. This provides a new perspective for studying the mutual predictive role of multiple factors and smartphone addiction. In practice, this study may provide some specific directions for the prevention and intervention of smartphone addiction among university students. It is important to focus on the important antecedents to the development of smartphone addiction in order to reduce the incidence of smartphone addiction, as well as focusing on the core influences in order to implement effective interventions when smartphone addiction occurs [27, 89].

### Limitations and future research directions

The main limitations of this study are as follows: firstly, this study used a questionnaire method for data collection, which is based on participants' self-reports, and there may be discrepancies between participants' reports of smartphone addiction with various related factors and the actual situation [90], making the questionnaire method somewhat subjective. In a follow up study, the present author will design experiments to further explore the relationship between smartphone addiction and related factors. Secondly, in terms of measuring smartphone addiction, the present author chose the Smartphone Addiction Scale for University Students, which is commonly used by Chinese researchers. The use of local smartphone scales may not be directly compared to the findings in other countries. In future research, the present author will select some scales that are popular internationally and also applicable in China, such as Smartphone Application Based Addition Scale (SABAS) [4, 6, 91, 92]. Thirdly, the present author used longitudinal data for network analysis to reveal causal predictive relationships between variables, but this study only involves data from two points in time, which does not provide a good indication of whether there is time-specificity in the predictive roles of the variables, and a longer tracking study should be conducted in the future. Fourthly, although the present author identified the important role of certain core influences on university students' smartphone addiction through network analysis, there is a lack of clinical evidence that interventions on these core influences are more effective than other interventions, and future research could test the effectiveness of programs that intervene on the core influences of smartphone addiction through clinical controls.

Despite the above limitations, this study provides a new perspective for exploring the relationship between university students' smartphone addiction and related influencing factors, and proposes possible directions for intervention in university students' smartphone addiction. In the future, research on university students' smartphone addiction based on network analysis will mainly include the following aspects. Firstly, network comparative testing will be used to compare the differences in network structure of smartphone addiction and its influencing factors among various demographic variables. For instance, cross-gender comparisons will be undertaken on university students' smartphone addiction and its influencing factors. Secondly, applying bridge network analysis to explore the comorbidity relationship between university students' smartphone addiction and other mental illnesses, such as post-traumatic stress disorder [93]. Finally, moderate network analysis can be used to test the effectiveness of specific intervention measures on university students' smartphone addiction [94].

## Conclusions

Self-control, academic burnout, social anxiety, and smartphone addiction have a mutually predictive relationship, both as antecedents to the formation of smartphone addiction and also as consequences of its creation. The core influences on smartphone addiction include self-control, self-esteem, academic burnout, and perceived social support. Future prevention and intervention for smartphone addiction should focus on these influences, which may be more cost-effective than other means.

### Supplementary Information

**Additional file 1: Appendix Fig. A1.** The cross-lagged panel network estimations of smartphone addiction and its influencing factors among university students from T1 to T2.

## Data Availability

The raw data supporting the conclusions of this article will be made available by the corresponding author, without undue reservation.
