# Supplementary material for: The unique role of smartphone addiction and related factors among university students: a model based on cross-sectional and cross-lagged network analyses
Source: BMC Psychiatry. 2023 Nov 27;23:883. doi: 10.1186/s12888-023-05384-6 (PMC10683260; doi:10.1186/s12888-023-05384-6)
Supplement: Supplementary file 1 — Additional file 1: Appendix Fig. A1. The cross-lagged panel network estimations of smartphone addiction and its influencing factors among university students from T1 to T2. [file 12888_2023_5384_MOESM1_ESM.pdf]

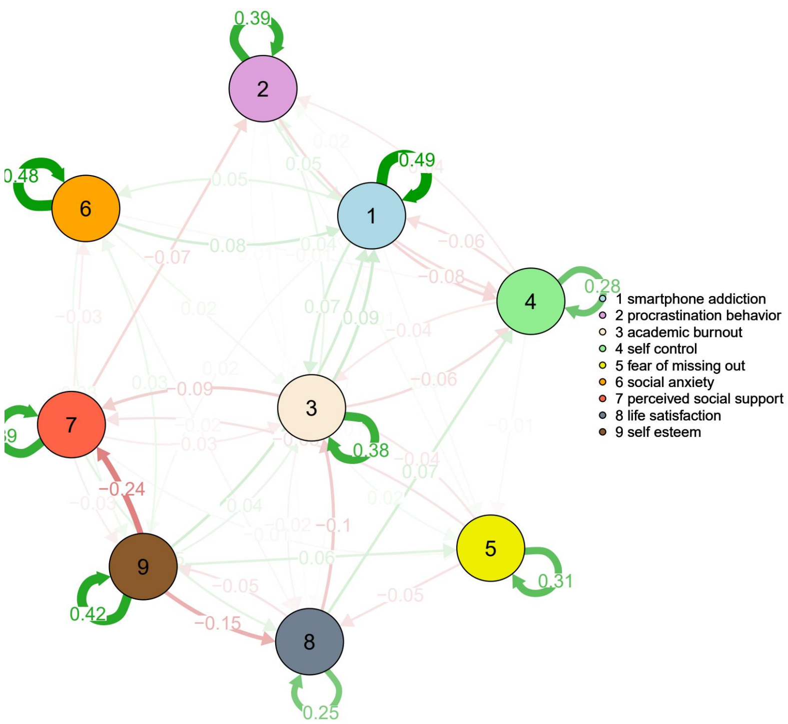

Appendix Fig A1. The cross-lagged panel network estimations of smartphone addiction and its influencing factors among university students from T1 to T2.
